# Vaginal microbial ecology in abnormal uterine bleeding: compositional alterations and pathophysiological impact as early predictors of secondary infection

**DOI:** 10.3389/fcimb.2026.1899052

**Published:** 2026-10-30

**Authors:** Ge Ge, Wen Xing, Jianshan Yuan, Beibei Fei

**Affiliations:** Department of Obstetrics and Gynecology, The Second Affiliated Hospital of Soochow University, Suzhou, Jiangsu, China

**Keywords:** abnormal uterine bleeding, influencing factors, morphology, vaginal infection, vaginal microecological function, vaginal microecological mechanism

## Abstract

**Background:**

Abnormal uterine bleeding (AUB) is a common gynecological condition that may disrupt the vaginal acidic environment and self-cleaning mechanisms, thereby predisposing patients to secondary vaginal infections. However, the predictive value of routine morphological and functional parameters for subsequent infection remains unclear.

**Objective:**

To study the current status of vaginal microecology morphology and function in patients with AUB, and to analyze its impact on secondary infections and its significance for early warning.

**Methods:**

A prospective study included 128 AUB patients (April 2021–April 2023) without initial vaginal infection. Vaginal secretions were collected for morphological and functional analysis. Patients were followed for 6 months and divided into infected (n=51) and non-infected (n=77) groups. Comparisons and ROC curve analysis were performed to evaluate predictive value.

**Results:**

In the infected group, vaginal bacteria concentration was higher. Proportions of abnormal pH, H_2_O_2_ secretion, positive leukocyte esterase, high Nugent score, and more leukocytes in vaginal secretions were higher, while the proportion of gram - positive bacteria was lower. Vaginal microbiota density grade I + IV, abnormal pH, H_2_O_2_ secretion, positive leukocyte esterase, Nugent score, and leukocyte count were positively correlated with secondary infection, and gram - positive bacteria were negatively correlated. The combined prediction of secondary infection had an AUC of 0.902, with 88.24% sensitivity and 76.62% specificity.

**Conclusion:**

The vaginal microecology of AUB patients is unbalanced, and the incidence of secondary infection is high, which may be related to the concentration of vaginal flora I+IV, the dominant bacteria being Gram-positive bacteria, abnormal H_2_O_2_ secretion, Nugent score, abnormal pH value, positive leukocyte esterase, and leukocyte count in vaginal secretions. The combined prediction of secondary infection of patients has certain reference value.

## 1 Introduction

Abnormal uterine bleeding (AUB) is one of the most common gynecological complaints worldwide (Lebduska et al., 2023). AUB is defined as uterine bleeding that deviates from normal menstruation in frequency, regularity, volume, or duration. According to the FIGO PALM-COEIN classification system, AUB is categorized into structural causes—including polyps, adenomyosis, leiomyoma, malignancy and hyperplasia—and non-structural causes—including coagulopathy, ovulatory dysfunction, endometrial factors, iatrogenic causes, and those not otherwise classified (Munro et al., 2018). The global prevalence of AUB ranges from 4% to 52%, with approximately half of cases showing no identifiable organic pathology. Beyond its immediate impact on quality of life, AUB poses significant long-term risks, including iron-deficiency anemia, infertility, and endometrial malignancy (Lebduska et al., 2023).

The vaginal microenvironment is a complex ecosystem critical to reproductive health (Dubé-Zinatelli et al., 2024). Under physiological conditions, the vaginal microbiota is dominated by Lactobacillus species, which maintain homeostasis through multiple mechanisms: production of lactic acid sustaining an acidic pH (3.5–4.5), secretion of hydrogen peroxide and bacteriocins with direct antimicrobial activity, adherence to epithelial cells forming a protective biofilm, and modulation of local immune responses (Aroutcheva et al., 2001). A recent narrative review highlighted the importance of differential production of lactic acid and its D/L isoforms, as well as bacteriocins and other core proteins produced by vaginal Lactobacillus spp., in maintaining vaginal health. Disruption of this ecosystem—characterized by Lactobacillus depletion, overgrowth of anaerobes, increased microbial diversity, and elevated pH—has been implicated in bacterial vaginosis, vulvovaginal candidiasis, pelvic inflammatory disease, and preterm birth (Cheng et al., 2025).

Emerging evidence has increasingly linked vaginal microbiome dysbiosis to AUB. The vaginal and uterine microbiomes are interconnected along a microbiota continuum rather than existing as isolated ecosystems (Chen et al., 2017). Importantly, AUB and vaginal infection risk appear to be bidirectionally related. Prolonged menstrual blood exposure (pH ~7.4) can neutralize the vaginal acidic environment and provide nutrients for pathogens such as Gardnerella and Prevotella, while vaginal dysbiosis-induced inflammation may disrupt endometrial hemostasis and exacerbate bleeding. Clinical evidence supports this association: a study of 372 patients with adenomyosis found a bacterial vaginosis prevalence of 19.4%, with menorrhagia rates significantly higher in those with bacterial vaginosis (Valdés-Bango et al., 2024). Similarly, among 189 patients with uterine fibroids, those with bacterial vaginosis had higher rates of abnormal uterine bleeding (65.5% vs. 26.3%, P < 0.001) and greater odds of submucosal fibroids (Robbins et al., 2024).

Beyond these clinical observations, recent large-scale epidemiological studies have further quantified the burden of AUB in specific populations. A meta-analysis of adolescent girls reported a pooled AUB prevalence of 19% (95% CI, 15.0–22.0%), with oligomenorrhea, metrorrhagia, and menorrhagia as the predominant subtypes (Krishna et al., 2026). In Ethiopia, 36.5% of reproductive-age women were found to experience AUB, with heavy menstrual bleeding and intermenstrual bleeding being the most frequent patterns (Derbew et al., 2025). Additionally, characterization of the lower genital tract microbiome in patients with benign gynecological diseases has revealed distinct compositional differences, further supporting the link between microbial dysbiosis and abnormal bleeding (Zhang et al., 2026).

Despite these findings, critical knowledge gaps persist. The clinical utility of routinely available morphological and functional parameters—including flora density, Nugent score, pH value, and hydrogen peroxide secretion—for predicting infection risk in AUB patients remains unevaluated. Prospective longitudinal data are scarce, and the predictive value of combining multiple indicators has not been explored. Furthermore, while 16S rRNA sequencing studies have characterized microbial composition differences, the practical application of simple, cost-effective microscopy-based parameters for early risk stratification in routine clinical settings has not been established.

Therefore, this prospective cohort study was designed to evaluate the predictive value of combined morphological and functional vaginal microecological parameters for early warning of secondary infections in AUB patients.

## 2 Methods

### 2.1 Subjects

A prospective study was conducted on 128 AUB patients admitted to our hospital from April 2021~April 2023, aged 26–50 years, with an average age of (36.38 ± 5.51) years. The continuous bleeding time was 7~30 days, with an average of (15.68 ± 4.31) days. The disease duration was 9~40 months, with an average of (24.25 ± 6.74) months; Body mass index was 19.2~25.8 kg/m2, with an average of (22.02 ± 1.51) kg/m2. The endometrial thickness was 7~14 mm, with an average of (10.89 ± 1.45) mm. The number of births was 1~4 times, and the average number of births was (2.20 ± 0.48) times.

The sample size was estimated using PASS software (version 15.0, NCSS, LLC, USA) based on a two-sided significance level of α = 0.05 and a power of 80%. Assuming a 30% difference in secondary infection rates between patients with abnormal versus normal vaginal microecology (based on pilot data), a minimum of 116 patients was required. Considering a 10% dropout rate, we aimed to enroll at least 128 patients, and all completed the 6-month follow-up with no loss to follow-up.

Baseline characteristics were compared between the infected group (n=51) and the non-infected group (n=77) at the end of the 6-month follow-up. There were no statistically significant differences between the two groups in terms of age, body mass index, disease duration, continuous bleeding duration, endometrial thickness, or parity (all P > 0.05), ensuring comparability of baseline characteristics (**Table 1**).

**Table 1:** Baseline characteristics comparison between infected and non-infected groups.

Characteristic	Infected group (n=51)	Non-infected group (n=77)	Statistic	P value
Age (years), mean ± SD	36.72 ± 5.48	36.15 ± 5.53	t = 0.571	0.569
BMI (kg/m²), mean ± SD	22.15 ± 1.49	21.93 ± 1.53	t = 0.805	0.422
Disease duration (months), mean ± SD	24.53 ± 6.81	24.06 ± 6.70	t = 0.384	0.702
Bleeding duration (days), mean ± SD	15.88 ± 4.42	15.55 ± 4.25	t = 0.421	0.674
Endometrial thickness (mm), mean ± SD	11.02 ± 1.41	10.80 ± 1.48	t = 0.844	0.400
Parity (times), mean ± SD	2.24 ± 0.49	2.17 ± 0.47	t = 0.815	0.417
Continuous bleeding days, median (IQR)	16.0 (12.0–19.0)	15.0 (11.0–18.0)	U = 0.893	0.372

Continuous variables were compared using independent-samples t-test for normally distributed data (age, BMI, disease duration, bleeding duration, endometrial thickness, parity) and Mann–Whitney U test for non-normally distributed variables (continuous bleeding days). All P values are two-sided.

### 2.2 Inclusion and exclusion criteria

Inclusion Criteria: AUB meets the diagnostic criteria of the Guidelines for the Diagnosis and Treatment of Abnormal Uterine Bleeding (Gynecologic Endocrinology Subgroup, Chinese Society of Obstetrics and Gynecology, Chinese Medical Association, 2014); Confirmed by uterine curettage or hysteroscopy and ultrasound; All are married women (this criterion served as a proxy for sexual activity, as pelvic examinations are not routinely performed on unmarried women in our setting, and sexual activity may affect vaginal microecology); No sexual history, vaginal medication or irrigation 3 days before vaginal microecology examination; Specimen collection is non-menstrual period; None of the patients had vaginal infection at the time of enrollment, and no fungal, trichomoniasis, or bacterial pathogenic microorganisms were detected in the vaginal discharge at the time of collection.

Exclusion Criteria: Malignancy; Accompanied by severe depression, anxiety and other mental illnesses; autoimmune diseases; intellectual and language dysfunction; Pregnant females; Uterine bleeding caused by drug-induced, systemic diseases, etc.; Severe heart, liver, kidney and other organ dysfunction; Hematologic dysfunction or bleeding disorders; infectious, acute and chronic infectious diseases; Use of broad-spectrum antibiotics, genital tract topical probiotics, or vaginal suppositories in the past 1 month; Presence of gynecological diseases such as pelvic inflammatory disease, endometritis, cervical erosion, etc.; Intrauterine implantation of a contraceptive device.

### 2.3 Methods

#### 2.3.1 Reagents and Equipment

Vaginal secretions were collected using disposable sterile swabs (Zhejiang Gongdong Medical Technology Co., Ltd., China). Gram staining was performed using the Gram Stain Kit (Thermo Scientific™ Remel™, R40080, USA), and morphological evaluation was conducted under an Olympus CX41 microscope (Olympus Corporation, Japan). For functional testing, pH value was measured using pH indicator strips (range 3.8–5.4, Hangzhou Diagreat Biotechnology Co., Ltd., China); H_2_O_2_ secretion was measured using a colorimetric assay kit (RayBiotech, ENZ4-BV-20, USA); and leukocyte esterase was measured using dry-chemistry test strips (specific for leukocyte esterase, Beijing Zhongsheng Jinyu Diagnostic Technology Co., Ltd., China), all following the manufacturer’s instructions. Sample processing involved vortex mixing and centrifugation in Eppendorf Safe-Lock microcentrifuge tubes (1.5 mL, Eppendorf, 0030120086, Germany).

#### 2.3.2 Specimen collection

Vaginal secretions were collected from the posterior vaginal fornix using two disposable sterile swabs, each rotated for 10–20 s and then removed. One swab was immediately used to prepare a direct smear on a clean glass slide for morphological evaluation. The second swab was placed into a sterile tube containing 0.5 mL of sterile normal saline for functional testing. All specimens were collected during the non-menstrual period, as stated in the inclusion criteria. However, we did not systematically record menstrual cycle length or estimate the specific cycle phase at the time of sampling. Although menstruation was excluded to avoid the acute interference of menstrual blood, we recognize that hormonal variations across the cycle may affect vaginal microbial composition and function. This factor was not controlled for in the present analysis, and we acknowledge it as a limitation of our study.

#### 2.3.3 Sample processing and storage

Following collection, one swab was immediately used for direct smear preparation on a clean glass slide for morphological evaluation. The second swab was placed into a sterile tube containing 0.5 mL of sterile normal saline and transported to the laboratory within 1 hour at 4 °C. Upon arrival, the tube was vortexed for 10 seconds to elute the secretions, and the swab was discarded. The eluate was centrifuged at 2000 rpm (approximately 400 × g) for 7 minutes at 4 °C. The supernatant was aliquoted for functional testing (pH, H_2_O_2_, leukocyte esterase), which was completed within 2 hours of collection. For long-term storage, functional test supernatants were preserved at −80 °C until further analysis, and repeated freeze-thaw cycles were avoided.

#### 2.3.4 Morphological testing

Saline wet mounts were prepared by mixing a small amount of vaginal secretion with normal saline on a glass slide, covered with a coverslip, and examined under a microscope (Olympus CX41, Japan) for the detection of pathogenic microorganisms such as Trichomonas vaginalis, budding spores, and hyphae. Gram-stained smears were prepared separately on clean slides, air-dried, heat-fixed, and stained using the Gram Stain Kit (Thermo Scientific™ Remel™, R40080, USA), then examined under oil immersion (×100) for the assessment of flora density, diversity, dominant bacteria, and Nugent score. Flora density and diversity were determined by microscopic examination under oil immersion (×100) using standardized four-grade criteria, rather than by next-generation sequencing. For Nugent scoring, the Gram-stained smears were evaluated according to the standardized method of Nugent et al. (1991): under oil immersion (×100), three bacterial morphotypes, large Gram-positive bacilli (Lactobacillus morphotypes), small Gram-negative or Gram-variable bacilli (Gardnerella/Bacteroides morphotypes), and curved Gram-negative or Gram-variable bacilli (Mobiluncus morphotypes), were semi-quantitatively scored from 0 to 4 each, yielding a total score of 0–10. Evaluation criteria (Cooperative Group of Infectious Disease, Chinese Society of Obstetrics and Gynocology, Chinese Medical Association, 2016): (1) Criteria for judging dominant bacteria: microorganisms seen the most under the microscope are defined as dominant bacteria; The dominant bacteria in the normal vagina are gram-positive bacilli, and the abnormal dominant bacteria include gram-negative bacilli, gram-positive cocci and gram-positive vibrio. (2) Vaginal flora density: judged by the average number of bacteria in each field of view. Grade I: 1~9; Grade II: 10~99; Grade III: ≥ 100; Grade IV: Bacteria clump together or densely cover mucosal epithelial cells. (3) Diversity of vaginal flora: judged according to the type of bacteria. Grade I: 1~3 kinds of bacteria; Grade II: 4~6 kinds of bacteria; Grade III: 7~10 kinds of bacteria; Grade IV: ≥ 11 species of bacteria. (4) Nugent score: 0~3 is normal, 4~6 is intermediate bacterial vaginosis (BV), and ≥7 points can be diagnosed as BV. (5) Donders score: 0~2 is normal, 3~4 is mild aerobic vaginitis (AV), 5~6 is moderate AV, and >6 is severe AV. (6) White blood cell count of vaginal discharge (pcs/high power field): The white blood cell count is abnormally elevated, indicating the presence of inflammation in the body.

#### 2.3.5 Functional testing

The functional testing procedures were performed according to the consensus of the Cooperative Group of Infectious Disease, Chinese Society of Obstetrics and Gynecology, Chinese Medical Association (2016). The second swab, designated for functional assays, was immediately placed into a sterile tube containing 0.5 mL of sterile normal saline and vortexed for 10 seconds to elute the vaginal secretions. All functional tests were performed within 2 hours of collection at room temperature, and the same trained technician operated all procedures using reagents from the same lot to ensure consistency.

1. pH value: A pH indicator strip (range 3.8–5.4, specifically designed for vaginal fluid) was dipped into the eluate, and the color change was read after 10 seconds by comparison with the manufacturer’s color chart. A pH within 3.8–4.5 was recorded as normal, and pH values <3.8 or >4.5 were considered abnormal.

2. H_2_O_2_ secretion: Hydrogen peroxide concentration in the eluate was measured using a colorimetric assay kit based on the peroxidase-catalyzed oxidation of a chromogenic substrate. After adding the reagent, the absorbance was read at 450 nm on a microplate reader, and concentrations were calculated from a standard curve run in parallel. A value ≥ 2 μmol/L was defined as H_2_O_2_ positive (indicating normal Lactobacillus function), while < 2 μmol/L was considered abnormal.

3. Leukocyte esterase: A commercially available dry-chemistry test strip specific for leukocyte esterase was immersed in the eluate, and the resulting color change was evaluated after 5 minutes against the manufacturer’s reference chart. Results were scored as negative or positive.

#### 2.3.6 Diagnostic criteria for vaginal microbiome

Normal vaginal microecology (Cooperative Group of Infectious Disease, Chinese Society of Obstetrics and Gynecology, Chinese Medical Association, 2021): no pathogenic microorganisms were found, the density and diversity of vaginal flora were grade II. ~ III., the function of lactobacillus was normal, the pH value was 3.8~4.5, the dominant bacteria were lactobacillus, leukocyte esterase, and sialidase were all negative. If any one of the above items is abnormal, the diagnosis is microbiome imbalance.

#### 2.3.7 Vaginal infection

(1) BV: Nugent score ≥ 7 points; (2) Trichomonas vaginalisis: Trichomonas vaginalis is seen under microscopic examination; (3) Vulvovaginal candidiasis: sporozoa or hyphae under microscopic examination; (4) AV: Donders>2 points; (5) Mixed vaginitis: vaginal inflammation caused by ≥ two pathogenic microorganisms. The presence of any of these items confirms the diagnosis of vaginal infection (Wang et al., 2023).

### 2.4 Outcome measure

A 6-month follow-up was chosen because AUB is a chronic condition; shorter intervals would not adequately capture the cumulative infection risk after repeated bleeding episodes, whereas 6 months aligns with routine clinical surveillance practice. Secondary infection was defined as any newly diagnosed vaginal infection (as per criteria in 2.3.5) occurring at any time during the 6-month follow-up, including both scheduled and unscheduled visits.

(1) The morphological and functional results of vaginal microecosystem in 128 patients were statistically analyzed. (2) After 6 months of outpatient follow-up, no cases were lost to follow-up, and the patients were divided into infected group (n=51) and non-infected group (n=77) according to whether there was secondary vaginal infection, and the morphological and functional test results of the vaginal microecosystem were compared between the two groups. (3) Spearman/Pearson analysis analyzed the correlation between the morphology and function of the vaginal microecosystem and secondary infection in patients. (4) To analyze the value of vaginal microecosystem morphology and function in predicting secondary infection.

### 2.5 Statistical analysis

Statistical analyses were performed using SPSS 25.0 and R 4.2. Continuous variables were first tested for normality using the Shapiro–Wilk test. Normally distributed data are presented as mean ± SD and were compared using the independent-samples t-test; non-normally distributed variables (e.g., Nugent score and leukocyte count) are expressed as median (IQR) and were compared using the Mann–Whitney U test. Categorical variables were compared using the χ² test (or Fisher’s exact test when appropriate). For correlation analysis, Spearman’s rank correlation coefficient was used for non-normally distributed and ordinal variables.

Variables with P < 0.10 in univariate analyses were entered into a multivariate binary logistic regression model using forward stepwise (likelihood ratio) selection. Variables with variance inflation factor (VIF) > 10 were considered collinear and were excluded to avoid multicollinearity; among correlated variables, the one with the strongest univariate association (lowest P value) was retained for the final model. To balance model parsimony with clinical comprehensiveness, the number of predictors was limited to a maximum of 7 based on the events-per-variable (EPV) rule, which recommends at least 5–10 events per predictor; with 51 infection events in this cohort, an EPV of approximately 7.3 (51/7) was considered acceptable. The original data were randomly divided into a training set (60%), a validation set (20%), and a test set (20%). The training set was used for model development; the validation set was used for hyperparameter tuning and internal validation; and the test set was reserved for final model evaluation. Model discrimination was assessed by the area under the ROC curve (AUC). For the combined prediction model, the predicted probability [Logit(P)] was used as the test variable to generate the ROC curve, and the optimal cut-off was determined by the Youden index. All tests were two-sided, and P < 0.05 was considered statistically significant.

The overall study design and workflow are summarized in **Figure 1**.

**Figure 1:**
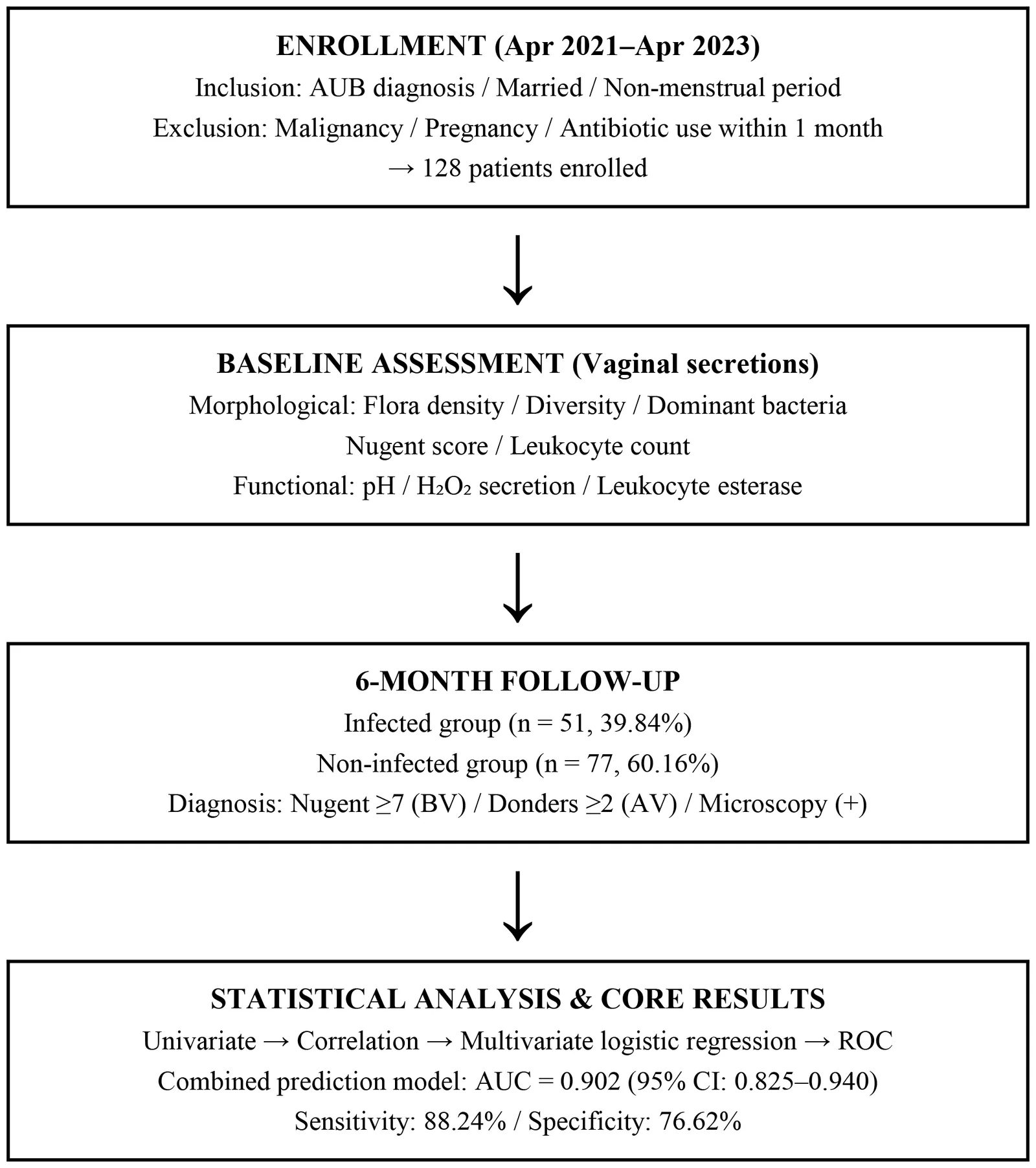
Study flowchart. AUB, abnormal uterine bleeding; BV, bacterial vaginosis; AV, aerobic vaginitis; ROC, receiver operating characteristic; AUC, area under the curve.

## 3 Results

### 3.1 Morphological and functional results of vaginal microecosystem in AUB patients

Among the 128 AUB patients, the density of vaginal microbiota and the diversity of vaginal microbiota were mainly grade II and III. 61.72% of the patients had gram-positive bacilli, Nugent score, Donders score, and mean white blood cell count of vaginal secretions were within the normal range, 39.06% of patients had abnormal pH value, 26.56% of patients had abnormal H_2_O_2_ secretion, and 93% of patients had positive leukocyte esterase with only 7.0% negative. Both sialidase and acetylglucosaminidase were negative. See **Table 2**.

**Table 2:** The morphological and functional results of vaginal microecosystem in 128 cases of AUB.

Parameter	Value
Morphological parameters	
Vaginal flora density, n (%)	
Grade I, IV	16 (12.5)
Grade II, III	112 (87.5)
Vaginal flora diversity, n (%)	
Grade I, IV	31 (24.2)
Grade II, III	97 (75.8)
Dominant bacteria: Gram-positive bacilli	79 (61.7)
Nugent score	2.97 ± 0.38
Donders score	1.01 ± 0.14
Leukocyte count (per high-power field)	6.06 ± 1.28
Functional parameters	
pH value	
≤ 4.5 (normal)	78 (60.9)
> 4.5 (abnormal)	50 (39.1)
H_2_O_2_ secretion	
≥ 2 μmol/L (normal)	94 (73.4)
< 2 μmol/L (abnormal)	34 (26.6)
Leukocyte esterase	
Positive	119 (93.0)
Negative	9 (7.0)

### 3.2 Comparison of the morphological and functional results of vaginal microecosystem between the two groups

After 6 months of follow-up, there were no lost follow-up cases, 128 AUB patients, 51 (39.84%) secondary infection, and 77 (60.16%) no infection. The proportion of patients with vaginal microbiota density I and IV., abnormal pH, abnormal H_2_O_2_ secretion, leukocyte esterase positive and Nugent score, and white blood cell count of vaginal secretions in the infected group were higher than those in the non-infected group, and the proportion of patients with gram-positive bacilli was less than that in the non-infected group (*P<0.05*). See **Table 3**.

**Table 3:** Comparison of the morphological and functional results of vaginal microecosystem between the two groups.

Vaginal microbiome	Infected group(n=51)	Non-infected group(n=77)	*t*/*χ^2^*/*u*	*P*
Morphological parameters				
Vaginal flora density			6.375	0.012
I, IV	11 (21.57)	5 (6.49)		
II, III	40 (78.43)	72 (93.51)		
Vaginal flora diversity			1.246	0.264
I, IV	15 (29.41)	16 (20.78)		
II, III	36 (70.59)	61 (79.22)		
Dominant bacteria: Gram-positive bacilli	25 (49.02)	54 (70.13)	5.787	0.016
Nugent score	4.05 ± 0.38	2.26 ± 0.49	22.053	<0.001
Donders score	1.03 ± 0.14	0.99 ± 0.12	1.727	0.087
Leukocyte count (per high-power field)	9.00 ± 1.28	4.12 ± 0.67	28.167	<0.001
Functional parameters				
pH value			66.747	<0.001
Normal	9 (17.65)	69 (89.61)		
Abnormal	42 (82.35)	8 (10.39)		
H_2_O_2_ secretion			30.242	<0.001
Normal	24 (47.06)	70 (90.91)		
Abnormal	27 (52.94)	7 (9.09)		
Leukocyte esterase			7.639	<0.001
Normal	43 (84.31)	76 (98.70)		
Abnormal	8 (15.69)	1 (1.30)		

### 3.3 Correlation of vaginal microecosystem morphology and function with secondary infection of AUB

Correlation analysis showed that vaginal microbiota density I.+IV. (rho = 0.223, P < 0.001), abnormal pH (rho = 0.683, P < 0.001), abnormal H_2_O_2_ secretion (rho = 0.457, P < 0.001), positive leukocyte esterase (rho = 0.209, P < 0.001), Nugent score (rho = 0.527, P < 0.001), and white blood cell count of vaginal discharge (rho = 0.611, P < 0.001) were positively correlated with secondary infection, while the dominant bacterium being gram-positive bacilli (rho = -0.236, P < 0.001) was negatively correlated with secondary infection (Spearman’s rho, all P < 0.001).

### 3.4 The morphological and functional values of vaginal microecosystem in predicting secondary infection of AUB

The ROC curve showed that the AUC of vaginal microbiota density I.+IV., dominant bacteria were gram-positive bacilli, abnormal pH, abnormal H_2_O_2_ secretion, positive leukocyte esterase, Nugent score, and white blood cell count of vaginal discharge were 0.616, 0.602, 0.860, 0.719, 0.572, 0.748, and 0.800, respectively, among which the specificity of leukocyte esterase positive was the highest, and the sensitivity of abnormal pH value was the highest, as shown in **Figure 2**, **Table 4**.

**Figure 2:**
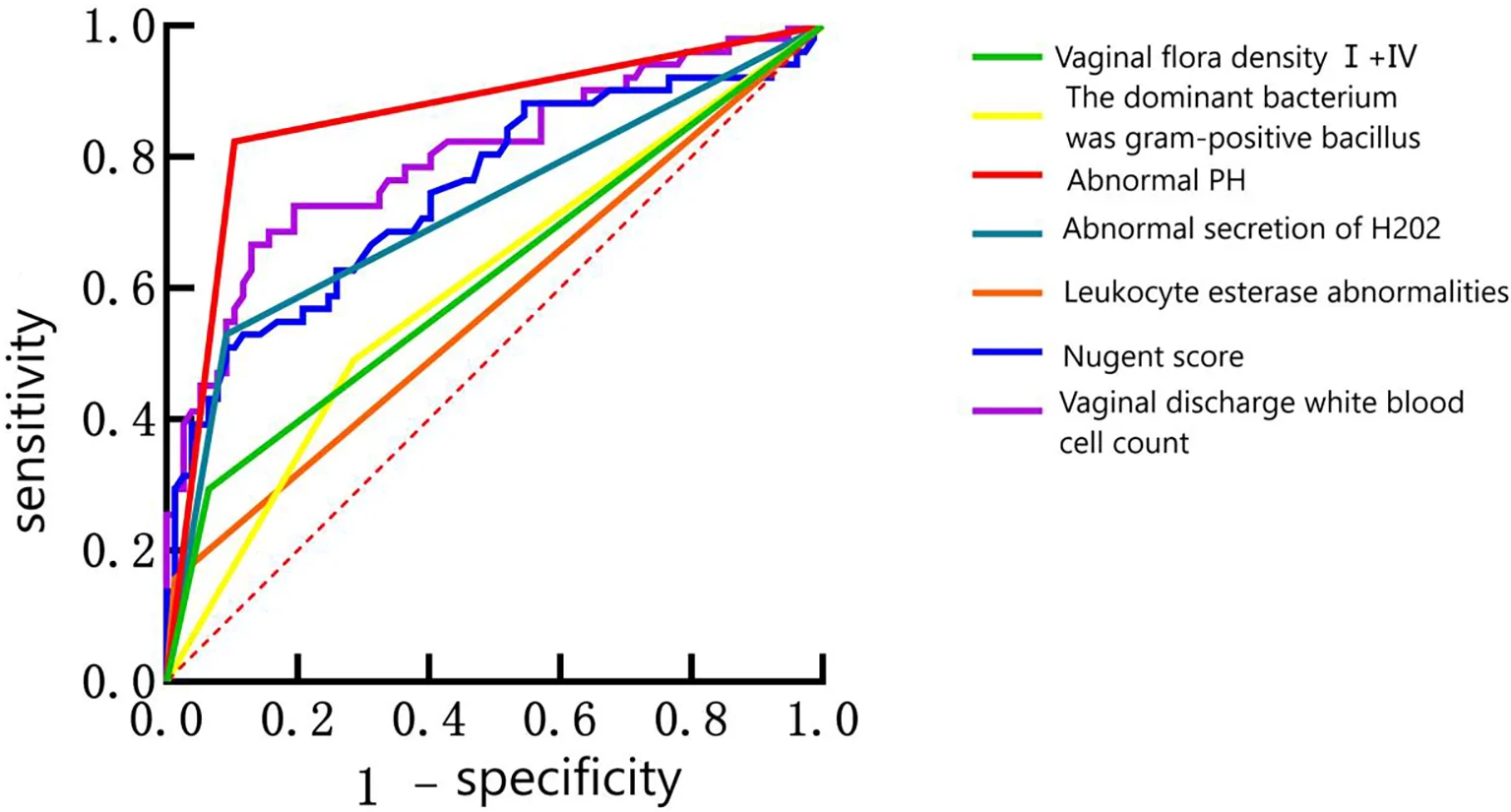
Morphology and functionalism of the vaginal microecosystem on ROC curves for secondary infection with AUB.

**Table 4:** Morphology and function of vaginal microecosystem for predicting ROC analysis of secondary infection with AUB.

Index	AUC	95% CI	Cut-off value	Sensitivity(%)	Specificity(%)	*P*
Vaginal flora density I.+IV	0.616	0.525∼0.699	I or IV Grade	29.41	93.51	<0.001
Dominant bacteria: Gram-positive bacilli	0.602	0.512∼0.688	Gram-positive bacilli	49.02	71.43	<0.001
Abnormal pH value	0.860	0.787∼0.915	Abnormal	82.35	89.61	<0.001
Abnormal H_2_O_2_ secretion	0.719	0.633∼0.795	Abnormal	52.94	90.91	<0.001
Leukocyte esterase abnormalities	0.572	0.481∼0.659	Positive	15.69	98.70	<0.001
Nugent score	0.748	0.663∼0.820	>4	50.98	90.91	<0.001
Vaginal discharge white blood cell count	0.800	0.720∼0.866	>8/high power field of view	66.67	87.01	<0.001

### 3.5 The value of the combination of morphology and functionology of the vaginal microecosystem in secondary infection of AUB

The final multivariate model was developed using forward stepwise logistic regression. Of the variables that met the univariate screening threshold (P < 0.10), Donders score was not retained in the final model because its predictive contribution was weaker than that of Nugent score and the two were highly correlated; Nugent score was selected as the more representative indicator of vaginal dysbiosis in this cohort. The final model included the following seven predictors: vaginal microbiota density I.+IV., dominant bacteria (Gram-positive bacilli), abnormal pH, abnormal H_2_O_2_ secretion, positive leukocyte esterase, Nugent score, and white blood cell count in vaginal secretions.

The original data were randomly divided into a training set (60%), a validation set (20%), and a test set (20%). The training set was used for model development; the validation set was used for hyperparameter tuning; and the test set was reserved for final model evaluation. The model achieved an AUC of 0.902 (95% CI: 0.825–0.940), with a sensitivity of 88.24% and a specificity of 76.62% (P < 0.05). See **Figure 3**.

**Figure 3:**
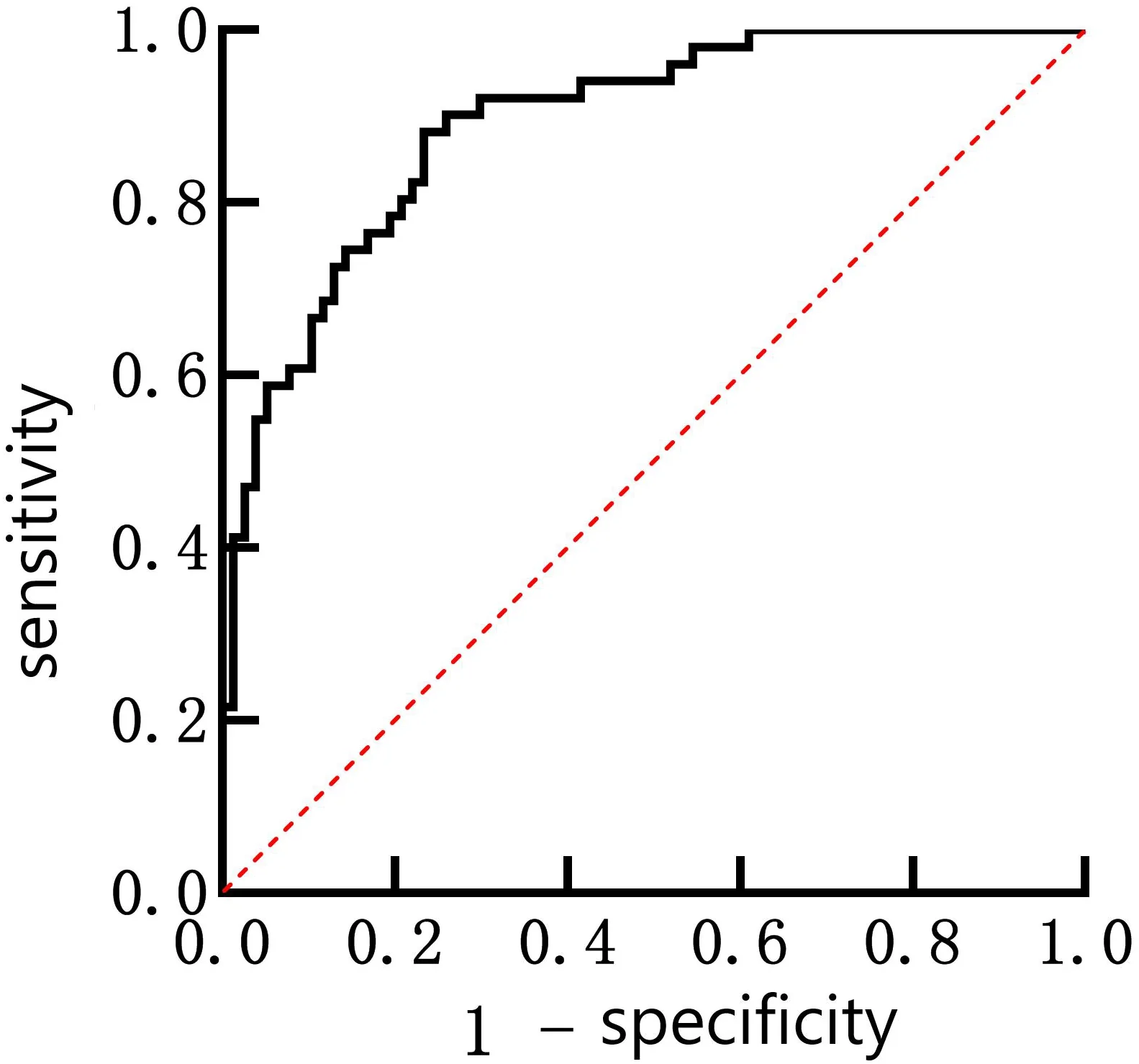
Morphology of vaginal microecosystem combined with functional prediction of ROC curves of secondary infection with AUB.

**Table 5** presents the multivariate logistic regression results for the final prediction model. Among the seven predictors, abnormal pH (OR = 4.82, 95% CI: 2.15–10.78, P < 0.001), Nugent score (OR = 2.56, 95% CI: 1.42–4.61, P = 0.002), and leukocyte count (OR = 2.13, 95% CI: 1.28–3.56, P = 0.004) were independently associated with secondary infection.

**Table 5:** Multivariate logistic regression analysis of predictors for secondary infection in AUB patients.

Predictor	β	SE	Wald χ²	OR	95% CI	P value
Vaginal flora density I.+IV.	0.85	0.42	4.10	2.34	1.03–5.32	0.043
Dominant bacteria: Gram-positive bacilli	−0.92	0.38	5.86	0.40	0.19–0.84	0.016
Abnormal pH	1.57	0.41	14.66	4.82	2.15–10.78	<0.001
Abnormal H_2_O_2_ secretion	0.68	0.39	3.04	1.97	0.92–4.22	0.081
Positive leukocyte esterase	0.45	0.52	0.75	1.57	0.57–4.33	0.386
Nugent score	0.94	0.30	9.80	2.56	1.42–4.61	0.002
Leukocyte count (per HPF)	0.76	0.26	8.54	2.13	1.28–3.56	0.004

HPF, high-power field; OR, odds ratio; CI, confidence interval. Variables with P < 0.05 were considered statistically significant.

### 3.6 Time-to-event analysis of secondary infection

Among the 128 patients, 51 (39.84%) developed secondary vaginal infection during the 6-month follow-up. The median time to infection was 10.5 weeks (interquartile range, 6.0–16.0 weeks). The Kaplan-Meier infection-free survival curve demonstrated a gradual decline in the probability of remaining uninfected over time, with the most pronounced decrease observed within the first 12 weeks (**Figure 4**). At the end of the 6-month follow-up, the infection-free survival rate was 68%, which directly corresponds to the cumulative infection incidence of 32% (1 − 0.6016). This time-dependent pattern further supports the association between baseline vaginal microecological abnormalities and the subsequent risk of infection.

**Figure 4:**
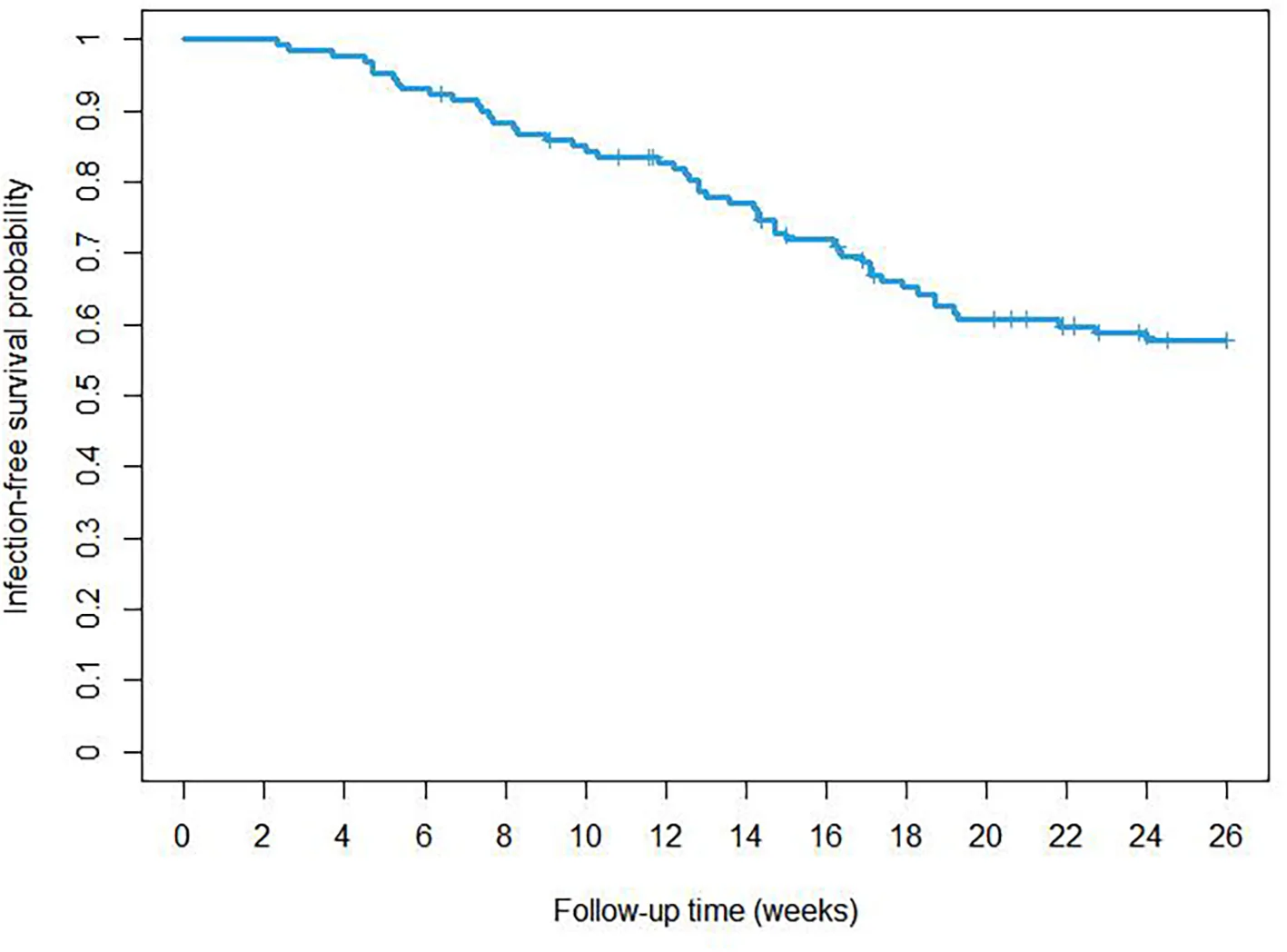
Kaplan-Meier curve of infection-free survival among AUB patients (N = 128) during the 6-month follow-up. The y-axis represents the probability of remaining free of secondary vaginal infection. The median time to infection was 10.5 weeks (IQR 6.0–16.0). At 6 months, the infection-free survival rate was 68%, corresponding to a cumulative infection incidence of 32%.

## 4 Discussion

Our findings demonstrate that AUB patients exhibit significant vaginal microecological imbalance, with a 32% incidence of secondary vaginal infections during 6-month follow-up. The combined morphological and functional prediction model—incorporating vaginal microbiota density I.+IV., dominant bacteria (Gram-positive bacilli), abnormal pH, abnormal H_2_O_2_ secretion, positive leukocyte esterase, Nugent score, and leukocyte count—achieved an AUC of 0.902 (95% CI: 0.825–0.940), with 88.24% sensitivity and 76.62% specificity, suggesting that routine vaginal microecological assessment may facilitate early risk stratification in this population.

Consistent with our results, emerging 16S rRNA sequencing studies have consistently shown that AUB patients exhibit altered vaginal microbial composition, characterized by increased alpha diversity, reduced Lactobacillus abundance, and a higher prevalence of community state type IV (CST IV) compared to healthy controls (Jimenez et al., 2024). Our observation of decreased gram-positive bacilli (primarily Lactobacillus) dominance in the infected group aligns with these sequencing-based findings. Another study reported that the microbiome profile is significantly different between patients with AUB and fibroids compared to those without AUB. Furthermore, patients with fibroids and abnormal bleeding exhibited higher abundance of BV-associated bacteria, including Gardnerella vaginalis, Megasphaera, Anaerococcus, Atopobium, Sneathia, and Mobiluncus (Shi et al., 2025).

The vaginal and uterine microbiomes are interconnected along a microbiota continuum rather than existing as isolated ecosystems. Chen et al. systematically sampled the female reproductive tract and identified distinct microbial communities in the cervical canal, uterus, fallopian tubes, and peritoneal fluid, reflecting a microbiota continuum along the female reproductive tract (Chen et al., 2017). More importantly, Wang et al. demonstrated that translocation of vaginal bacteria into the uterine cavity modulates endometrial health—transplanting certain strains of vaginal bacteria into the vagina of rats induces or reduces endometritis-like symptoms, verifying the interdependent relationship between vaginal bacterial translocation and uterine microecology (Wang et al., 2021). Building on this framework, our prospective data provide clinical evidence that AUB-associated vaginal microecological disruption may predispose to secondary infections, potentially through a microbiota continuum mechanism that extends beyond the vagina to the upper reproductive tract.

The mechanistic link between AUB and secondary vaginal infections appears bidirectional. Sustained or excessive menstrual bleeding (pH ~7.4) can neutralize the normal acidic vaginal environment (pH 3.5–4.5), impairing self-cleaning mechanisms and facilitating pathogenic overgrowth (Kaur et al., 2020). Lactobacillus species—the keystone taxa of the healthy vaginal microbiota—maintain homeostasis through production of lactic acid (sustaining pH 3.5–4.5), secretion of hydrogen peroxide (H_2_O_2_) and bacteriocins, adherence to epithelial cells, and modulation of local immune responses (Tachedjian et al., 2017; France et al., 2022). Depletion of Lactobacillus and reduced H_2_O_2_ production, as observed in our infected group, compromise these protective barriers (Witkin et al., 2007; Ravel et al., 2011). Additionally, vaginal dysbiosis-induced inflammation, characterized by elevated pro-inflammatory cytokines including IL-1α, IL-1β, IL-6, IL-8, and MCP-1, may disrupt endometrial hemostasis and exacerbate bleeding (Melis et al., 2015; Aniewski et al., 2018). This creates a vicious cycle: bleeding promotes dysbiosis, and dysbiosis-associated inflammation may worsen bleeding.

Clinical evidence further supports this association. Valdez-Sinon et al. conducted a retrospective study of 372 patients with adenomyosis and found a BV prevalence of 19.4%, with menorrhagia rates significantly higher in those with BV (P < 0.01), demonstrating a positive association between menorrhagia and BV episodes (Valdez-Sinon et al., 2025). Cheng et al. reported that among 189 patients with uterine fibroids, those with BV had higher rates of abnormal uterine bleeding (65.5% vs. 26.3%, P < 0.001) and greater odds of submucosal fibroids (OR: 3.0, 95% CI: 1.3–7.1) (Cheng et al., 2023). These findings corroborate our observation that AUB patients are at elevated risk for subsequent vaginal infections and highlight the clinical relevance of routine microecological surveillance.

Several limitations of this study should be acknowledged. First, this was a single-center prospective study with a relatively modest sample size, which may limit the generalizability of our findings. Second, although we excluded patients with baseline vaginal infections at enrollment, we did not systematically record detailed symptom-based clinical assessments—such as changes in discharge (volume, color, consistency), intense vulvar pruritus, erythema, dysuria, dyspareunia, or prior use of over-the-counter antifungals—during follow-up. Vaginal infections were diagnosed using standardized laboratory criteria (Nugent score, Donders score, and microscopic examination) rather than comprehensive clinical evaluation guidelines, which may have led to under-recognition of mild or subclinical infections. Third, we did not control for menstrual cycle phase at the time of specimen collection, and hormonal variations across the cycle may influence vaginal microbial composition and functional parameters. Fourth, although Donders score was assessed at baseline, it was not included in the correlation, ROC, or multivariate analyses. This was because it showed only a marginal difference between groups and exhibited high collinearity with Nugent score, which was retained as the more representative indicator of vaginal dysbiosis in this cohort given its stronger univariate association with secondary infection. Future studies incorporating standardized clinical assessment tools—such as the Cleveland Clinic Vaginitis Guide or similar validated protocols—alongside longitudinal microbiome profiling are warranted to further validate and extend our predictive model.

In summary, the imbalance of vaginal microbiota in AUB patients can increase the secondary infection rate of patients, which may be related to the patient’s vaginal microbiota density I.+IV., the dominant bacteria are gram-positive bacilli, abnormal pH, abnormal H_2_O_2_ secretion, Nugent score, positive leukocyte esterase, and white blood cell count of vaginal secretions.

## Data Availability

The original contributions presented in the study are included in the article/Supplementary Material. Further inquiries can be directed to the corresponding author.
